# Radiotherapy for High-Risk Non-Muscle-Invasive Bladder Cancer: Current Evidence and Future Directions

**DOI:** 10.3390/curroncol33040225

**Published:** 2026-04-17

**Authors:** Lucas Resende Salgado, Osama Zaytoun, Ahmed Rabie, Nicholas Murphy, Anthony Nehlsen, Kristin Hsieh, Zachary Dovey, Anum Aamir, Kunal K. Sindhu

**Affiliations:** 1 Department of Urology and Radiology, Mount Sinai, New York, NY 10029, USA; lucas.resendesalgado@mountsinai.org (L.R.S.); nicholas.murphy@mountsinai.org (N.M.); anthony.nehlsen@mountsinai.org (A.N.); kristin.hsieh@mountsinai.org (K.H.); zachary.dovey@mountsinai.org (Z.D.); anum.aamir@mountsinai.org (A.A.); kunal.sindhu@mountsinai.org (K.K.S.); 2 Department of Urology, Northwell Health, New York, NY 11042, USA

**Keywords:** bladder cancer, NMIBC, radiotherapy, chemoradiotherapy, bladder preservation

## Abstract

**Introduction:** Non-muscle-invasive bladder cancer (NMIBC) represents approximately 78% of newly diagnosed bladder cancers and is characterized by high recurrence rates and variable progression risk. While transurethral resection of bladder tumor (TURBT) followed by intravesical therapy remains standard management, optimal treatment of high-risk and Bacillus Calmette-Guerin (BCG)-unresponsive disease remains controversial. Radiotherapy (RT), particularly in combination with chemotherapy, has been explored as a bladder-preserving alternative. **Material and Methods:** We conducted a narrative review of published literature evaluating the role of definitive RT in high-risk NMIBC, with emphasis on T1 disease. Retrospective series, prospective trials, meta-analyses, and contemporary guideline recommendations were examined. For each included study, we extracted data on the extent of TURBT (maximal vs. incomplete/non-specified), use and type of concurrent chemotherapy, radiotherapy technique (3D-conformal, IMRT, or proton), treatment volume (bladder only vs. whole pelvis), and dose/fractionation schedule. **Results:** Early studies evaluating RT alone demonstrated modest complete response rates. More recent approaches incorporating maximal TURBT followed by concurrent chemoradiotherapy report improved outcomes, with complete response rates of approximately 80–88% and 5-year overall survival comparable to surgical series. The phase II NRG/RTOG 0926 trial in recurrent high-risk T1 disease after intravesical therapy failure demonstrated an 81% complete response rate and favorable bladder preservation outcomes. Meta-analytic data suggest 5-year recurrence-free survival around 54% and overall survival near 70%, although evidence remains limited and largely non-randomized. Advances in image-guided and hypofractionated RT may further improve therapeutic outcomes while limiting toxicity. **Conclusions:** while definitive chemoradiotherapy is a promising option for selected patients, it remains investigational and should be considered only in those who are unfit for or decline radical cystectomy. Prospective randomized studies are needed to better define its role in contemporary management.

## Simple Summary

Radiotherapy for high-risk non-muscle-invasive bladder cancer (NMIBC) has historically been supported by limited, heterogeneous data, leaving its role undefined in guidelines. Existing evidence—largely retrospective series and one phase II trial—suggests that maximal transurethral resection followed by concurrent chemoradiotherapy can achieve complete response rates of approximately 80% and 5-year overall survival comparable to cystectomy in selected patients. Our review synthesizes these data and highlights two key findings: first, whole-pelvis radiotherapy may offer a survival advantage over bladder-only treatment, based on a recent meta-analysis in muscle-invasive bladder cancer; second, emerging trials are now testing hypofractionated radiotherapy, immunotherapy combinations, and biomarker-driven selection specifically in NMIBC. These findings underscore the need for prospective, randomized studies to define optimal target volumes, fractionation, and integration with novel systemic agents, ultimately moving radiotherapy from an investigational salvage option toward an established bladder-preserving standard for well-selected patients.

## 1. Introduction

Bladder cancer is a global health priority, ranking as the ninth most common malignancy with approximately 614,000 new cases and 220,000 deaths annually [1]. In the United States, nearly 85,000 cases and 17,000 deaths occur each year, with disproportionate mortality observed in Black patients [2,3,4,5]. While urothelial carcinoma (UC) constitutes > 90% of cases in the West, geographic variations—such as *Schistosoma*-endemic regions—influence the prevalence of squamous cell carcinoma [6,7,8].

Approximately 75% of patients present with non-muscle-invasive bladder cancer (NMIBC) (stages Ta, Tis, and T1), characterized by high recurrence rates (up to 78%) and variable risk of progression to muscle-invasive disease (up to 45%) [9,10,11]. High-risk NMIBC—defined by T1 tumors, carcinoma in situ (CIS), or large/multifocal high-grade disease—is primarily managed with transurethral resection (TURBT) followed by intravesical Bacillus Calmette–Guérin (BCG) [12,13]. However, many patients become BCG-unresponsive or intolerant. For these individuals, radical cystectomy is the standard of care but carries significant morbidity and impacts quality of life [14,15].

The etiology of bladder cancer is multifactorial; tobacco smoking remains the primary modifiable risk factor, though chronic inflammation, chemical exposures, and prior radiation also contribute [16,17,18]. Molecularly, tumors diverge into indolent pathways (mutations in *FGFR3* and *HRAS*) or aggressive, high-grade pathways (alterations in *TP53*, *RB1*, and ERBB2) [19,20,21]. Typically presenting with painless hematuria, the diagnosis is confirmed via cystoscopy and TURBT [22].

Given the resource-intensive nature of NMIBC management and the need for lifelong surveillance, bladder-preserving alternatives are urgently required for those unfit for or declining surgery [23,24,25].

Recent evidence, such as the NRG Oncology/RTOG 0926 trial, suggests that definitive radiotherapy (RT) can achieve high rates of bladder preservation in selected high-risk NMIBC patients [26,27]. This review summarizes the oncologic evidence for RT in high-risk NMIBC, focusing on treatment techniques, dose fractionation, and the integration of emerging technologies like immunotherapy and image guidance.

## 2. Treatment Approaches for Muscle and Non-Muscle-Invasive Bladder Cancer

The current management of NMIBC is guided by risk stratification established by the American Urological Association (AUA) (Table 1), which categorizes patients into low-, intermediate-, and high-risk groups based on tumor stage, grade, size, multifocality, recurrence history, and presence of carcinoma in situ (CIS) [21,22]. High-risk NMIBC includes high-grade T1 tumors, CIS, multifocal or large (>3 cm) high-grade Ta/T1 disease, BCG-unresponsive status, lymphovascular invasion, variant histology, and prostatic urethral involvement [21,22].

Initial diagnosis and treatment begin with transurethral resection of bladder tumor (TURBT), which is both diagnostic and therapeutic. A single immediate postoperative intravesical dose of gemcitabine or mitomycin C (MMC) is routinely given to reduce recurrence, particularly in low-risk disease [23,24,25]. Adjuvant therapy is dictated by risk category. For high-risk NMIBC, the standard of care after TURBT is intravesical Bacillus Calmette–Guérin (BCG) induction followed by maintenance therapy, which has been shown to improve recurrence-free survival [27,28,29,30]. However, many patients develop BCG-unresponsive disease, defined as persistent or recurrent high-grade NMIBC within 12 months of adequate BCG therapy [31,32,33,34].

For patients with BCG-unresponsive disease who are fit for surgery, radical cystectomy remains the standard recommendation [35,36,37,38]. Nevertheless, cystectomy carries significant morbidity and impacts quality of life [39]. In patients who are unfit for or decline cystectomy, bladder-preserving alternatives are needed. Options include intravesical chemotherapy (gemcitabine or MMC), systemic immunotherapy (pembrolizumab), gene therapy (nadofaragene firadenovec), or a novel interleukin-15 agonist combined with BCG [40]. In selected cases, definitive radiotherapy with concurrent radiosensitizing chemotherapy has also been explored as a bladder-preserving approach, extrapolated from the established role of trimodality therapy in muscle-invasive bladder cancer [41,42,43].

Although the present review focuses on NMIBC, the rationale for using chemoradiotherapy in high-risk NMIBC is derived largely from the established role of trimodality therapy in muscle-invasive bladder cancer (MIBC). In MIBC, randomized trials have demonstrated that maximal TURBT followed by concurrent chemoradiotherapy achieves bladder preservation rates of 60–80% with overall survival comparable to radical cystectomy in selected patients [42,43]. Additional prospective studies and meta-analyses have further refined optimal chemotherapy partners (cisplatin and 5-fluorouracil + mitomycin C) and radiotherapy dose/fractionation schedules (conventional 60–66 Gy or hypofractionated 55 Gy in 20 fractions) [44,45,46,47,48,49]. While these MIBC data are not directly applicable to NMIBC, they provide the biological and clinical foundation for investigating similar approaches in high-risk, BCG-unresponsive NMIBC, as discussed in the following sections.

## 3. Current Standard of Care for High-Risk NMIBC

There is a paucity of evidence analyzing the role of RT in high-risk T1 bladder cancer. In fact, the data for the role of radiation therapy in this setting comes primarily from retrospective studies. Few prospective studies have examined this question.

Early data pertaining to the use of RT in T1 bladder cancer originated in the early 1980s. A series from 1986 examining patients treated with definitive radiation therapy (doses ranging from 55 to 57.5 Gy) for T1 bladder cancer showed complete response rates of ~50% [38]. A subsequent retrospective series from Princess Margaret, analyzing outcomes in 355 patients with bladder cancer treated with doses ranging from 47.5 to 52.25 Gy (almost a third of whom had T1 disease), reported a complete response rate of 69% and a local relapse-free survival rate of 35% at 10 years [39]. In contrast to the aforementioned institutions, a combined approach with both TURBT and chemoRT is utilized, even for high-risk T1 cancers, at the University of Erlanger in Germany. Clinicians there have reported markedly better outcomes with their paradigm, including a complete response rate of 88% and a local control rate of 61% at 5 years. The overall and disease-specific survival for this cohort at 5 years was 71% and 81%, respectively, which are comparable to utilizing cystectomy as primary management. Finally, 80% of patients were able to preserve their bladder with this approach [40,41]. In this series, treatment was commenced by TURBT aimed at maximal, complete resection (if feasible) of the tumor mass. Residual tumor was assessed histologically by biopsies from all resection margins. Additional evaluation included chest radiography and computed tomography of the abdomen and pelvis. RT was initiated 4 to 6 weeks after initial TURBT using 6- to 10-MV photons and a four-field box technique with individually shaped portals and daily fractions of 1.8 to 2 Gy on five consecutive days. A median dose of 55.8 Gy (range, 45.0 to 61.4 Gy) was applied to the bladder. Pelvic nodes were irradiated with a median dose of 50.4 Gy (range, 36.0 to 54.1 Gy). Since October 1985, chemotherapy has been given simultaneously for five consecutive days during the first and fifth weeks of RT. Forty-three patients received cisplatin 25 mg/m^2^/d; in 16 patients with decreased creatinine clearance (<50 mL/min), carboplatin 65 mg/m^2^/d was administered. Since 1993, 54 patients were treated with a combination of cisplatin or carboplatin and fluorouracil 600 mg/m^2^/d. A total of eight patients also received deep regional hyperthermia within an ongoing phase II study.

The most notable prospective randomized study in this population compared intravesical therapy versus definitive radiotherapy to doses of 60 Gy in patients with T1, grade 3 transitional cell carcinoma. The authors reported no difference in outcomes, but worse toxicity (both acute and late) from RT [41]. However, this study failed to utilize concurrent chemotherapy and reported generally poor outcomes as compared to historical data. As we now know from randomized trials examining patients with muscle-invasive bladder cancer, the addition of chemotherapy to radiation therapy reduces local failures as well as rates of cystectomy [42,43]. “The chemotherapy regimens used in NMIBC chemoradiotherapy studies have been adapted from MIBC protocols, though evidence specific to NMIBC remains limited. 

A 2021 meta-analysis examined the role of RT in patients with high-risk NMIBC. The authors included 13 studies with 746 patients. Most patients were male (87.2%), with a median age of 67.3 years. Nearly two-thirds received RT only, while the remaining approximately one-third received concurrent chemotherapy. While most patients received between 50 and 55 Gy to their bladders, total doses as low as 30 Gy and as high as 77 Gy were noted. Cystoscopies were the most common form of assessment for treatment response. The overall complete response rates to primary therapy, as reported by 8 studies, was 78.2%. In utilizing six studies, the meta-analysis concluded that the five-year recurrence-free survival rate for patients with high-risk NMIBC treated with RT was 54%, and the 5-year overall survival rate was 72% [45]. These results are comparable to those seen in patients treated with intravesical therapy [33,45,46,47]. However, given the overall paucity of supporting evidence, this meta-analysis concluded that while RT may offer some benefit, the data supporting it are limited and of poor quality.

The most recent prospective study examining the role of RT for patients with high-risk NMIBC came out in 2024. RTOG 0926 looked at bladder-preserving trimodality therapy for T1 bladder cancer [48]. This phase 2 study focused on patients with recurrent T1 bladder cancer after failing intravesical therapy who would have otherwise undergone cystectomy. Radiation therapy involved a total dose of 61.2 Gy in 34 daily fractions (approximately 7 weeks) using three-dimensional conformal techniques. Small pelvic field irradiation was applied to the entire bladder and prostatic urethra (in men), as well as the obturator and iliac lymph nodes (internal and external). A dose of 41.4 Gy in 23 fractions was delivered, followed by a sequential boost of 19.8 Gy in 11 fractions to the whole bladder, totaling 61.2 Gy.

Radiosensitizing chemotherapy included cisplatin, mitomycin, or 5-fluorouracil (5-FU), chosen by the treating physician. Cisplatin was administered 3 days/wk for 60 min during weeks 1, 3, and 5 of radiation therapy at a dose of 15 mg/m^2^/d. Patients receiving mitomycin/5-FU were treated with mitomycin 12 mg/m^2^ on day 1 and infused with 5-FU at 500 mg/m^2^/24 h for 5 days during weeks 1 and 4 of radiotherapy. Follow-up assessments were performed every 3 months for 1 year, every 4 months during year 2, every 6 months during years 3–5, and then annually thereafter.

This study showed that 30 out of 37 patients had a complete response (81%). Overall survival at 3 and 5 years were 69.5% and 56.4%, respectively, and a cancer-specific mortality of 25% at 5 years, comparable to large surgical series [49]. At 3 years, the cystectomy rate was roughly 12.4%. Although this was a small phase 2 study and larger studies are required to further evaluate alternatives to cystectomy for patients who fail intravesical therapy, chemoradiation after a maximum TURBT can be considered as an alternative for elderly patients or those with multiple co-morbidities precluding a cystectomy with non-muscle-invasive bladder.

Beyond the NMIBC-specific data summarized above, a recent systematic review and meta-analysis by [49] provides relevant insights into the optimal radiotherapy target volume. This meta-analysis included six studies with 3655 patients and compared bladder-only radiotherapy versus whole pelvis radiotherapy in patients with lymph node-negative muscle-invasive bladder cancer (MIBC) undergoing trimodality treatment. The findings demonstrated that whole pelvis radiotherapy was associated with significantly better overall survival (OR 0.76, 95% CI: 0.66–0.89, *p* = 0.0004) and improved cancer-specific survival (OR 0.70, 95% CI: 0.50–0.98, *p* = 0.04), with no significant difference in disease-free survival. A trend toward higher acute gastrointestinal toxicity was observed with whole pelvis radiotherapy (*p* = 0.06). Although this meta-analysis focused on MIBC, its findings inform the ongoing debate regarding elective nodal irradiation in high-risk NMIBC, where prospective data specifically for NMIBC are lacking [50]. A detailed summary of key studies, including study design, patient population, treatment details, and oncologic outcomes, is provided in Table 2.

## 4. Future Directions

Over the past few years, there has been a shift toward bladder-sparing therapies for patients with NMIBC. The role of radiotherapy continues to evolve, with several efforts exploring optimized delivery and combination strategies.

Hypofractionated radiotherapy—Based on non-inferiority data from muscle-invasive bladder cancer (MIBC) trials (e.g., 55 Gy in 20 fractions), hypofractionated schedules are now being tested in NMIBC. Ongoing trials such as TRAIN (BCG-naïve high-risk NMIBC) are evaluating hypofractionated radiotherapy with or without radiosensitizers [51].

Advanced radiotherapy techniques—Image-guided radiotherapy (IGRT) and intensity-modulated radiotherapy (IMRT) allow precise targeting of the bladder while reducing dose to adjacent organs (bowel, rectum, and femoral heads), potentially lowering acute and late toxicity. Image-guided radiotherapy (IGRT) and intensity-modulated radiotherapy (IMRT) are now standard techniques in many centers; however, prospective data specifically evaluating their impact on toxicity and outcomes in NMIBC are lacking. Emerging modalities such as proton beam therapy may further reduce dose to surrounding organs, although evidence in NMIBC remains limited [53,54]

Combining radiotherapy with immunotherapy—Preclinical and early clinical data suggest synergistic effects when radiotherapy is combined with immune checkpoint inhibitors. The phase II PARRC trial (NRG-GU014) is directly testing bladder radiotherapy plus chemotherapy versus radiotherapy plus pembrolizumab in patients with high-grade T1 NMIBC recommended for cystectomy [52]. Results are eagerly awaited.

Biomarker-driven patient selection—Molecular subtyping, gene expression profiles, and circulating tumor DNA may help predict which patients with high-risk NMIBC are most likely to respond to radiotherapy and achieve durable bladder preservation [55,56]. Large prospective validation studies are needed before these biomarkers can be integrated into routine clinical decision-making.

The available evidence, though limited, suggests that maximal TURBT prior to radiotherapy is associated with higher complete response rates, likely by removing all visible disease and leaving only microscopic residual tumor for radiotherapy to address, consistent with the MIBC trimodality paradigm. The addition of concurrent chemotherapy appears critical, as early studies using radiotherapy alone reported inferior outcomes. Regarding target volume, the optimal extent of radiotherapy (bladder only vs. whole pelvis) in high-risk NMIBC remains uncertain. Extrapolating from a recent meta-analysis in node-negative MIBC (Granda, 2025) [50], whole pelvis radiotherapy was associated with improved overall and cancer-specific survival, albeit with a trend toward increased acute gastrointestinal toxicity. In the absence of NMIBC-specific data, some investigators have suggested elective nodal irradiation for patients with very high-risk features (e.g., lymphovascular invasion and variant histology), though prospective data are lacking [57], while bladder-only treatment may suffice for well-selected patients with unifocal disease and no adverse features. Hypofractionated regimens (e.g., 55 Gy in 20 fractions) are increasingly used based on non-inferiority data from MIBC trials and offer convenience and potential radiobiological advantages.

In summary, future research should prioritize prospective randomized trials that define optimal radiotherapy dose/fractionation, target volumes (bladder-only vs. whole pelvis), and combination partners (chemotherapy, immunotherapy, or radiosensitizers) specifically for high-risk NMIBC.

## 5. Conclusions

The available evidence for definitive radiotherapy in high-risk NMIBC remains limited, consisting largely of retrospective series, one phase II trial (NRG/RTOG 0926), and a single meta-analysis. Despite these limitations, complete response rates of approximately 80% and 5-year overall survival of 56–72% have been reported, comparable to outcomes from radical cystectomy series in selected patients. Maximal TURBT followed by concurrent chemoradiotherapy appears to offer the best outcomes, with whole pelvis radiotherapy potentially providing a survival advantage over bladder-only treatment; however, the optimal radiotherapy target volume (bladder-only vs. whole pelvis) remains uncertain and requires prospective evaluation specifically in NMIBC.

However, given the paucity of high-level evidence, definitive chemoradiotherapy for high-risk NMIBC should be considered investigational and reserved for patients who are unfit for or decline radical cystectomy, ideally within a clinical trial or multidisciplinary setting. Ongoing prospective randomized trials (TRAIN, PARRC and BRACE) [51,52,58] will help clarify the role of radiotherapy in BCG-unresponsive disease, optimal target volumes, dose fractionation, and combination with immunotherapy.

Until such data emerge, the decision to offer radiotherapy for high-risk NMIBC requires careful patient selection, shared decision-making, and treatment at experienced centers with expertise in bladder-preserving trimodality therapy.

## Figures and Tables

**Table 1:** AUA/SUO risk stratification. AUA risk stratification for NMIBC.

Low Risk	Intermediate Risk	High Risk
Low-grade solitary Ta, ≤3 cm	Recurrence in 1 year, LG Ta	High-grade T1
Papillary urothelial neoplasm of low malignant potential	Solitary low-grade Ta > 3 cm	Any recurrent, High-grade Ta
	Low-grade Ta, multifocal	High-grade Ta, >3 cm (or multifocal)
	High-grade Ta, ≤3 cm	Any carcinoma in situ
	Low-grade T1	Any BCG failure in high-grade patient
		Any variant histology
		Any lymphovascular invasion
		Any high-grade prostatic urethral involvement

**Table 2:** Summary of key studies evaluating radiotherapy in high-risk NMIBC.

Study (Year)	Design	Population	Treatment Details	Key Oncologic Outcomes
Quilty & Duncan (1986) [38]	Retrospective	T1 bladder cancer	RT alone (55–57.5 Gy)	CR ~50%
Gospodarowicz et al. (1991) [39]	Retrospective	Mixed stages (≈1/3 T1)	RT alone (47.5–52.25 Gy)	CR 69%; 10-year local RFS 35%
Weiss et al. (2006, 2008) [40,41]	Retrospective	High-risk T1	Maximal TURBT + chemoRT	CR 88%; 5-year local control 61%; 5-year OS 71%; bladder preservation 80%
Harland et al. (2007) [42]	Prospective randomized	pT1G3	RT alone (60 Gy) vs. intravesical therapy	No difference in outcomes; worse toxicity with RT (no concurrent chemo)
Rodrigues Pessoa et al. (2021) [45]	Meta-analysis (13 studies, *n* = 746)	High-risk NMIBC	RT ± concurrent chemo (majority 50–55 Gy)	Pooled CR 78.2%; 5-year RFS 54%; 5-year OS 72%
Dahl et al. (NRG/RTOG 0926, 2024) [26]	Phase II (*n* = 37)	Recurrent high-grade T1 after intravesical therapy failure	Maximal TURBT + chemoRT (61.2 Gy/34 fractions; elective pelvic nodes RT treated)	CR 81%; 3-year OS 69.5%, 5-year OS 56.4%; 3-year cystectomy rate 12.4%
Granda (IJROBP, 2025) [50] *	Systematic review + meta-analysis (6 studies, *n* = 3655)	Lymph node-negative MIBC (cT2-T4N0) undergoing TMT—informs nodal irradiation debate in NMIBC	Bladder-only RT vs. whole pelvis RT (±chemotherapy)	WP-RT significantly improved OS (OR 0.76, *p* = 0.0004) and CSS (OR 0.70, *p* = 0.04); no difference in DFS; trend toward higher acute GI toxicity with WP-RT (*p* = 0.06)
TRAIN Trial (ISRCTN16345179; EAU 2026) [51]	Phase III RCT (*n* = 328)	BCG-naïve HR-NMIBC after maximal TURBT	RT 55 Gy/20 fractions + radiosensitizer vs. intravesical BCG	Primary endpoint: event-free survival; active recruiting
PARRC Trial (NRG-GU014; NCT06770582) [52]	Phase II RCT	High-grade T1 recommended for cystectomy	Bladder RT + chemotherapy vs. RT + pembrolizumab	Primary endpoint: bladder-intact event-free survival; active recruiting

* Although this meta-analysis focuses on MIBC, its findings on target volume (bladder-only vs. whole pelvis) are relevant to ongoing discussions about elective nodal irradiation in high-risk NMIBC. Abbreviations: CR, complete response; RFS, recurrence-free survival; OS, overall survival; CSS, cancer-specific survival; TMT, trimodality therapy; RCT, randomized controlled trial; HR, high-risk; TURBT, transurethral resection of bladder tumor; chemoRT, chemoradiotherapy; WP-RT, whole pelvis radiotherapy; bladder-only radiotherapy; GI, gastrointestinal; GU, genitourinary.

## Data Availability

No new data were created or analyzed in this study.
